# Microfluidic EPG recordings show striking pharyngeal pumping phenotype in a *C. elegans* Alzheimer’s disease model

**DOI:** 10.17912/W2WC7M

**Published:** 2016-10-21

**Authors:** Janis C. Weeks, Kristin J. Robinson, Benedicta Wanjeri, Philip F. Copenhaver, William M. Roberts

**Affiliations:** 1 Institute of Neuroscience, University of Oregon, Eugene OR 97403, USA; 2 NemaMetrix, Inc,. 44 W 7th Ave., Eugene, OR 97401 USA.; 3 Dept. of Cell, Developmental and Cancer Biology, Oregon Health & Science University, Portland, OR, 97239. USA

**Figure 1.**
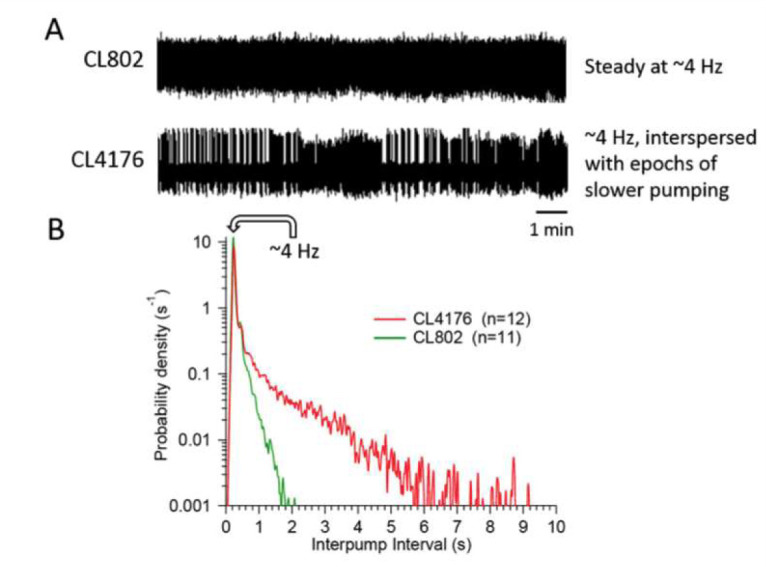


## Description

Strain CL4176 accumulates human amyloid-β1-42 in body wall muscles after animals are shifted from 15 to 25 oC, causing paralysis (Link et al. 2003). We temperature-shifted worms at early L4 and quantified pharyngeal pumping at 48 h using a microfluidic electropharyngeogram (EPG) recording platform (Lockery et al. 2012; Weeks et al. 2016). Recordings were made in M9 buffer with 10 mM serotonin and analyzed by automated pump-recognition software. At 48 h, control CL802 worms showed normal pumping activity (**A**) whereas the mean pump frequency in CL4176 worms was significantly decreased (**A**; CL802, 4.14 ± 0.11 Hz; CL4176, 2.98 ± 0.18 Hz; mean ± S.E.M.; *P* <10-5, 2-tailed Student’s t-test; 4 independent replicates). The decreased pump frequency was not a uniform slow-down but instead resulted from a striking increase in the probability of long inter-pump intervals (the time between successive pumps; **B,** mean ± S.E.M. shown by lines and shading). As seen in **B**, the normal modal pump frequency of ~4 Hz was still present in CL4176, but the probability of interruptions lasting several seconds was greatly increased.

Interestingly, the pumping phenotype preceded amyloid-β1-42-induced paralysis. The table in **C** shows the time course of paralysis after temperature up-shift. Some worms in the population were paralyzed at 48 h, but they were not used for EPG recordings. Recordings made 24 h post-shift were not analyzed in detail but appeared normal (data not shown).

In summary, disrupted pumping was an earlier marker than paralysis in CL4176 worms. The finding that pumping was perturbed even though amyloid-β1-42 was not expressed in pharyngeal muscle supports other findings that feeding provides a general readout of physiological health in *C. elegans*. The microfluidic EPG platform permits semi-automated collection of large quantities of data; e.g., a 15 min recording of a CL802 control worm contains ~3600 pumps, whereas visual counts of pharyngeal pumping are typically performed for only 10-60 s. The increased sensitivity and convenience provided by the microfluidic EPG platform may enhance research into amyloid-β toxicity in *C. elegans* Alzheimer’s disease models.
